# Recent advances in understanding neocortical development

**DOI:** 10.12688/f1000research.20332.1

**Published:** 2019-10-23

**Authors:** Victor Borrell

**Affiliations:** 1 Institute of Neuroscience, Consejo Superior de Investigaciones Científicas (CSIC) - Universidad Miguel Hernández, Ramon y Cajal s/n, 03550 San Juan de Alicante, Spain

**Keywords:** Radial Glia, cell lineage, OSVZ, cortex folding, basal progenitors, neurogenesis, human-specific neocortex, neocortical development, cerebral cortex evolution, progenitor proliferation

## Abstract

The neocortex is the largest part of the mammalian brain and is the seat of our higher cognitive functions. This outstanding neural structure increased massively in size and complexity during evolution in a process recapitulated today during the development of extant mammals. Accordingly, defects in neocortical development commonly result in severe intellectual and social deficits. Thus, understanding the development of the neocortex benefits from understanding its evolution and disease and also informs about their underlying mechanisms. Here, I briefly summarize the most recent and outstanding advances in our understanding of neocortical development and focus particularly on dorsal progenitors and excitatory neurons. I place special emphasis on the specification of neural stem cells in distinct classes and their proliferation and production of neurons and then discuss recent findings on neuronal migration. Recent discoveries on the genetic evolution of neocortical development are presented with a particular focus on primates. Progress on all these fronts is being accelerated by high-throughput gene expression analyses and particularly single-cell transcriptomics. I end with novel insights into the involvement of microglia in embryonic brain development and how improvements in cultured cerebral organoids are gradually consolidating them as faithful models of neocortex development in humans.

## Introduction

The cerebral cortex, or neocortex, is the part of our brains primarily responsible for abstract thinking and our unique cognitive abilities as human beings. It is by far the most complex biological structure, and it forms during embryonic development following a sequence of genetically predefined molecular and cellular events. This process of embryonic development recapitulates the emergence of the human cerebral cortex during evolution. Thus, understanding neocortical development in humans and other species tells us about the magnitude of its complexity, how it differs from neocortical development in non-humans, how it emerged during evolution from simpler brains, and what goes wrong in developmental brain disease.

Through the use of informative experimental animal species, much has been learned about the basic processes of neocortical development: neural stem and progenitor cell (NSPC) proliferation, generation, and migration of neurons from their place of birth to their final location in neocortical layers and the growth of neural processes and establishment of nerve connections. Recent advances in genome editing, tissue culture, and DNA sequencing have accelerated our understanding of neocortical development at many levels. Here, I briefly summarize some of the most significant advances in our understanding of neocortical development generated recently by the international community, and I give special attention to progenitor proliferation and neurogenesis of dorsal progenitors and projection neurons. Although much remains to be learned, the emerging picture shows that the evolutionary and developmental emergence of the human neocortex involved a plethora of genetic and epigenetic changes, including novel genes and variants, functional gene networks, and novel cell types and cellular specializations.

## Neurogenesis

Development of the neocortex begins with the proliferation of NSPCs, which amplify their own pool prior to generating neurons. Recent work shows that the proliferation of NSPCs and their mode of neuron production vary across species, and previously unsuspected mechanisms of molecular regulation have been identified. Novel insights about the diversity of progenitor cells and how their neuronal output contributes to build the cerebral cortex are also emerging.

### Progenitor cell diversity

Work in the last decade has identified a diversity of NSPC types and their lineage relationships. Following the seminal discovery of apical radial glial cells (aRGCs) as the primary type of cortical progenitor cell ^1, 2^, intermediate progenitor cells (IPCs) were identified ^3– 5^. IPCs are transit-amplifying progenitors expressing the transcription factor Tbr2 and producing the majority of excitatory neurons in the mouse and rat neocortex ^3, 4, 6, 7^. Most IPCs cluster and undergo mitosis in a layer basal from the ventricular zone (VZ), the subventricular zone (SVZ), although a subset reside in the VZ and undergo apical mitosis, named short neural precursors ^8^. Further studies in primates led to the discovery of an expanded and specialized SVZ, subdivided in inner and outer domains (ISVZ and OSVZ, respectively) ^9^. These layers were later identified in other species with an expanded neocortex, like ferret, cat, and sheep ^10^ and even in New World monkeys and the Amazonian rodent agouti ^11, 12^. Progenitor cell lineage tracings in human, macaque, ferret, and mouse led to the discovery of additional types of basal progenitor cells in the developing neocortex, which are bound by complex lineage relationships. These include various types of basal radial glial cells (bRGCs) ^10, 11, 13– 15^ and subapical progenitor cells ^16^. The relative abundance and proliferative capacity of these progenitor cell types are much greater in species with a neocortex that is large and folded (carnivores and primates) than in those where it is small and smooth (mouse and rat). This has been proposed to contribute to the evolutionary increase in complexity, or complexification **,** of the neocortex ^16– 18^.

Pioneer transcriptomic studies of the developing cerebral cortex using bulk tissue, identified differences in gene expression between germinal layers, cortical areas, developmental stages, and mammalian species ^15, 19– 23^. This illustrated the profound diversity of transcriptional landscapes in cortical development at all levels, including folding and across phylogeny, and set the conceptual foundations for the next technological leap: transcriptomics of single cells. The advent of single-cell transcriptomic analyses has revolutionized our approach to studying cortical development by providing a global and unbiased picture of cell diversity with unprecedented resolution ^24– 26^. This technology has enabled identification of multiple sets of transcriptionally distinct progenitor cell classes in the cortical primordium, generating excitatory neurons ^27– 29^, and in the basal ganglia, generating inhibitory interneurons ^30, 31^.

Single-cell analyses are also beginning to shed light on long-standing hypotheses about the heterogeneity of cortical progenitor cells and the dynamics of their lineage and fate potential during development ^32– 36^. Early experimental studies of these fundamental questions indicated that the fate potential of cortical progenitors is temporally restricted, such that early progenitors can produce neurons for all cortical layers but late progenitors can produce neurons for superficial layers only ^34, 37^. Such late fate restriction would be cell-autonomous as late cortical progenitors continued producing superficial-layer neurons even when transplanted into the new cellular environment of a young host cortex ^34, 37^. The identification of a subset of RGCs that expressed *Cux2* and that were fate-restricted to produce upper-layer neurons further supported this model ^35^. However, such fate-restricted progenitors have not been identified in single-cell transcriptomic studies ^28, 33, 38^. Rather, some of these studies support the existence of epigenetically regulated temporal molecular birthmarks in RGCs, which act in their daughter neurons as seeds for neuronal diversity. It is proposed that these conserved differentiation programs may then be integrated with environmental (non-cell-autonomous) cues to ultimately define the identity of the neuronal progeny ^28, 39, 40^. Nevertheless, controversial points of view on these and related issues remain because of differences in single-cell data processing, analysis, and interpretation ^30, 31, 41^.

### Modes of neurogenesis and influence on cortex size

Cortical excitatory neurons may be generated directly from the primary progenitor cells (aRGCs) or indirectly via secondary basal progenitors such as IPCs and bRGCs. Indirect neurogenesis is considered a milestone of mammalian cortical evolution, enabling a phenomenal increase in the numbers of neurons produced, particularly those destined to superficial layers, and leading to cortical expansion ^42, 43^. Recent studies reveal that indirect neurogenesis is the major mode of producing deep as well as superficial cortical layers in mouse ^40, 44^ and that it also exists in non-mammals, albeit at lower levels and mostly in birds ^44, 45^. At the molecular level, regulation of the balance between direct and indirect neurogenesis is phylogenetically conserved across amniotes, from snakes to birds and mammals, including humans, where high Robo1/2 signaling promotes expression of the Notch ligands Jag1/2 while repressing the canonical ligand Dll1 ^44^. Blockade of this signaling system drives indirect neurogenesis, increasing neuron numbers and cortex size. Intriguingly, indirect neurogenesis also correlates with hyperpolarization of apical progenitor cells, which represses Wnt signaling ^40^. Changes in the mode of neurogenesis are responsible for the reduced cortex size in developmental brain disease, like microcephaly induced by Zika virus infection. In this case, activation of the unfolded protein response drives direct neurogenesis, leading to premature and limited neuron production and to a small cortex ^46– 48^.

### Regulation of progenitor cell proliferation

Cerebral cortex size depends on the mode of neurogenesis and also on the proliferative activity of cortical progenitor cells. Multiple mechanisms regulating progenitor cell proliferation have recently been uncovered. Regulation of gene transcription by epigenetic mechanisms has emerged as a key factor where histone deacetylases and methyltransferases regulate the generation and position of IPCs, neuron migration, and cortical lamination ^49, 50^. Similarly, regulation of chromatin accessibility and other mechanisms related to non-coding genomic regions critically determines levels and patterns of gene expression in the developing cortex, defining neuron production, cortex size, and area identity ^51, 52^.

One of the most exciting findings has been the identification of mRNA species transported within the long basal process of aRGCs and locally translated at their basal endfeet, next to the pial membranes ^53– 55^. This includes proteins regulating the cell cycle, like Ccnd2, and lengthening of G _1_, S, and M phases of the cell cycle, which depletes progenitor cells causing premature neurogenesis or apoptosis ^56– 58^.

IPCs are usually depicted as round cells extending few short processes ^3, 59^, and bRGCs are usually depicted as having a single smooth and unbranched basal process ^10, 60– 62^. Recent observations demonstrate that the degree of process branching and elaboration in both IPCs and bRGCs is greater in ferret and human (which have a large and folded cortex) than in mouse (which has a small and smooth cortex) and this is linked to a greater proliferation rate of these cortical progenitor cells ^63, 64^. Basal progenitor process growth and proliferation are related to the membrane-bound protein PALMD, enabling these cells to receive pro-proliferative signals related to integrin function ^60, 64, 65^. Indeed, modulation of cortical progenitor cell proliferation by cell-extrinsic signals has now been demonstrated to occur from multiple sources, including growing axons and migrating neurons ^63, 66, 67^, extracellular neurotransmitters and ions ^68, 69^, and Notch, Wnt, Fgf, and Shh signals ^70^.

Once IPCs and neurons are born from aRGCs, they must first delaminate and migrate away from the VZ toward the SVZ. This critical process is regulated by several signaling mechanisms, including Robo ^71^, YAP ^72^, Insm1 via the apical adherens protein Plekha7 ^73^, and the centrosome-associated protein Akna ^74^.

## Neuronal migration

Once neurons are born, they must migrate from their place of birth in the germinal layers to their final location in the neuronal layers. Neuronal migration is a multi-step process that begins with neuronal delamination from the VZ and movement to the SVZ. (See Silva *et al*. ^67^ [2019] for an excellent review on neuron migration.)

### Delamination

New advances in understanding cortical development have identified the microtubule-associated protein Lszts as a master regulator of cellular dynamics, promoting the delamination of neurons and bRGCs born from aRGCs by altering apical junction organization ^75^.

### Multipolar phase transition

Once newborn neurons delaminate from the VZ, they enter the SVZ and undergo a transition phase displaying multipolar morphology ^76^. During this process, NeuroD1 expressed by multipolar cells represses Prdm16, which regulates mitochondrial reactive oxygen species, and this signaling axis is crucial for the regulation of the multipolar phase migration ^77^. Dbnl, a protein interacting with F-actin, is another key regulator of neuronal multipolar morphology, polarity, and migration by regulating the levels of N-cadherin ^78^.

### Bipolar locomotion

Cortical neurons resume radial migration by re-acquiring polarity and extending a single leading process as they exit the SVZ. This leading process establishes intimate adhesive interactions with the basal process of RGCs for guidance in their migratory displacement. The long-held concept that the leading process of these radially migrating neurons is single and unbranched ^79, 80^ has been challenged by new observations, demonstrating frequent branching and thus more complex migratory behaviors than previously reported ^81^. In fact, leading process branching is much more frequent in the developing cortex of ferret than mouse and is related to the tangential displacement of radially migrating neurons. Thus, this seems directly related to the maintenance of the radial organization of the cerebral cortex during the tangential expansion and folding of the neocortex ^81, 82^. However, an excessive tangential displacement of cortical neurons is deleterious, and the horizontal tiling of the developing cerebral cortex, or regular distribution of its radial units, must be actively maintained. The microtubule stability regulator protein Memo1 plays key functions in the maintenance of RGC structure and cortical tiling by repressing the hyperbranching of the basal process of RGCs and the excessive dispersion of radially migrating neurons ^83^. On the other hand, it was recently shown that the chromatin-modifying enzyme Prdm16 is also necessary for transcriptional silencing in RGCs and to promote the migration of late-born cortical neurons and cortical layering ^49^.

## Evolution of cerebral cortex development

A fundamental feature of the evolution of cerebral cortex in amniotes is the phenomenal increase in neuron number and expansion in size. This process is recapitulated during embryonic development, and recent work demonstrates the importance of the balance between direct and indirect neurogenesis ^44^. Mechanisms regulating this critical balance, including transcriptional programs regulated by progenitor cell membrane polarity ^40^ and canonical signaling pathways like the unfolded protein response ^46^, some of which are highly conserved across amniotes like Robo and Dll1 ^44^, are beginning to be identified.

Beyond the emergence of indirect neurogenesis, cortical evolution involved additional key mechanisms. Cell lineage labeling and single-cell transcriptional analyses have revealed a remarkable evolutionary increase in diversity of cortical progenitor cells, particularly at the genetic level. This includes, for example, multiple subtypes of aRGCs and bRGCs identified in human, macaque, or ferret but not in mouse or rat ^14, 15, 64, 84^. Likewise, innovations in progenitor cell lineages are critical for the emergence and expansion of the OSVZ ^15, 85^, the basal germinal layer typical of big and folded brains, which is absent in mice and is perturbed in human diseases that affect brain size ^85^.

The search for genetic mechanisms evolved in primate and human phylogeny which are likely relevant in the evolution of their neocortex, has led to the identification of primate-specific and human-specific genetic programs expressed in the developing cerebral cortex. These include whole collections of primate-specific miRNAs targeting cell cycle genes ^22^ and also miRNA-mRNA modules in the embryonic human brain that undergo dynamic transitions during development and that identify expression networks in specific cell types ^86, 87^. As for protein-coding genes, recent studies have identified genes that emerged in the recent human lineage by means of total or partial duplication, and that promote cortical progenitor cell proliferation. Other studies have identified programs of gene expression in cortical progenitor cells that are human-specific ^88– 91^.

Species with large brains have a tendency of being folded, and knowledge of specific mechanisms involved in cortex folding has also increased recently. This highlights the relevance of the sodium ion channel *SCN3A* function in progenitor cells ^68^, of gliogenesis ^92^, and of extracellular matrix proteins ^93^, in the mechanical aspect of tissue folding. Intriguingly, the organization of folds in the frontal cortex is widely conserved from Old World monkeys to hominoids ^94^, which offers a remarkable opportunity to study cortical evolution and the acquisition of higher brain functions in primates.

## Discovering the developmental importance of microglia

Largely ignored previously in the field of neocortical development, microglia have become a new relevant component in our understanding of this process. Microglial cells are found in the developing neocortex at much earlier stages and at much greater abundance than previously considered; there, they interact intimately with progenitor cells and regulate their number, hence emerging as important players in the regulation of neurogenesis ^95– 97^. Microglia may also contribute to progenitor cell delamination and expansion of ISVZ/OSVZ in primates ^95^. Moreover, microglia are critical regulators of brain wiring, contributing to the specification of neural circuits and relaying information from the periphery, including the microbiota ^98, 99^.

## Cerebral organoids as a model of human neocortical development

Although it is not possible to study the development of the human neocortex at the experimental level, major technological breakthroughs in the last few years on stem cell reprogramming and tissue culture offer possibilities that were previously unthinkable. Following the first protocol to generate cerebral organoids from human embryonic stem cells and induced pluripotent stem cells ^100^, these have become the Rosetta stone to study and manipulate human brain development ^101^. Not only can we now grow human cerebral organoids for many months, recapitulating many of the early features of cortical development, but they can be grown to form functional circuits and be responsive to sensory stimuli ^102^. Most importantly, the recent design of a culture protocol to generate highly reproducible cerebral organoids is a fundamental milestone for the consolidation of this as a faithful model of human brain development ^103^. Comparison between organoids grown from human and chimpanzee cells reveal human-specific features of cortical progenitor cells ^104^ and the validity of these organoids to advance our understanding of human brain evolution ^105, 106^.

## Conclusions

Understanding neocortical development requires working at many different levels, from single-cell transcriptomics to tissue mechanics, and this must be applied to studying histogenesis at multiple levels, from neurogenesis to connectivity and cortex folding. Understanding neocortical evolution and disease requires understanding development across relevant informative species and in the context of genetic or contextual failure. Recent technological advances offer unprecedented opportunities for conducting this research; for example, single-cell genomic analyses help elucidate molecular changes in human brain disease at the resolution of cell types ^107^, and patient-derived iPSCs are used to model and hopefully rescue the disease ^108^. Only through our ability to use and combine these amazing tools in creative ways will we decipher what makes us human and what genetic changes occurred during evolution that led to the development and emergence of the human neocortex.

## Abbreviations

aRGC, apical radial glial cell; bRGC, basal radial glial cell; IPC, intermediate progenitor cell; ISVZ, inner subventricular zone; NSPC, neural stem and progenitor cell; OSVZ, outer subventricular zone; RGC, radial glial cell; SVZ, subventricular zone; VZ, ventricular zone
